# Upregulation of Fibrinogen-Like 1 Expression Contributes to Reducing the Progression of Preeclampsia

**DOI:** 10.3389/fcell.2021.757643

**Published:** 2021-12-08

**Authors:** Tsung-Lin Cheng, Chung-Hwan Chen, Meng-Hsing Wu, Chao-Han Lai, Ko-Hung Lee, Sheng-Hsiang Lin, Ai-Li Shiau, Chao-Liang Wu, Lin Kang

**Affiliations:** ^1^ Department of Physiology, School of Medicine, College of Medicine, Kaohsiung Medical University, Kaohsiung, Taiwan; ^2^ Regenerative Medicine and Cell Therapy Research Center, Kaohsiung Medical University, Kaohsiung, Taiwan; ^3^ Orthopaedic Research Center, Kaohsiung Medical University, Kaohsiung, Taiwan; ^4^ Department of Orthopedics, Kaohsiung Municipal Ta-Tung Hospital, Kaohsiung Medical University, Kaohsiung, Taiwan; ^5^ Division of Adult Reconstruction Surgery, Department of Orthopedics, Kaohsiung Medical University Hospital, Kaohsiung Medical University, Kaohsiung, Taiwan; ^6^ Department of Obstetrics and Gynecology, National Cheng Kung University Hospital, College of Medicine, National Cheng Kung University, Tainan, Taiwan; ^7^ Department of Biochemistry and Molecular Biology, College of Medicine, National Cheng Kung University, Tainan, Taiwan; ^8^ Department of Surgery, National Cheng Kung University Hospital, College of Medicine, National Cheng Kung University, Tainan, Taiwan; ^9^ An-an Women and Children Clinic, Tainan, Taiwan; ^10^ College of Medicine, Institute of Clinical Medicine, National Cheng Kung University, Tainan, Taiwan; ^11^ Department of Microbiology and Immunology, College of Medicine, National Cheng Kung University, Tainan, Taiwan

**Keywords:** fibrinogen-like 1, preeclampsia, placenta, embryo (animal), proinflammatory cytokines

## Abstract

Fibrinogen-like 1 (FGL1) is involved in liver injury and liver regeneration, but its role in placenta and preeclampsia (PE) remains unclear. We assessed FGL1 expression in serum and placenta from L-NAME-induced PE-like mouse and in women with (*n* = 38) and without (*n* = 42) PE. For the mouse study, pregnant C57Bl/6 mouse (*n* = 6/group) were subcutaneously administered L-NAME with or without FGL1 once daily starting on days 7–14 of pregnancy and were sacrificed on gestational day (GD) 20. Maternal body weight, blood pressure, and urinary protein were assessed during GDs 8–20. The weight and length of the placenta and fetus were assessed. The placental structure was evaluated using hematoxylin staining. In the human study, the sera of the pregnant women during the late trimester were assessed with enzyme-linked immunosorbent assays (ELISAs). FGL1 expression in human trophoblast cell lines under L-NAME stimulation was measured using Western blotting and immunofluorescence staining. The detected FGL1 protein levels in serum and placenta were both significantly upregulated in patients and mouse with PE compared with those in the non-PE groups. FGL1 treatment decreased maternal hypertension and proteinuria, decreased fetal weight in mouse with PE, downregulated proinflammatory cytokine (interleukin-1b and interleukin-6) levels, and maintained the balance between antiangiogenic (fms-like tyrosine kinase-1) and proangiogenic (placental growth factor) substances in the placenta. L-NAME-upregulated FGL1 expression was inhibited following overexpression of FoxO3a. In summary, FoxO3a reduction is a potential pathophysiological mechanism leading to upregulated placental FGL1 expression that may play a pivotal role in preventing PE progression.

## Introduction

Preeclampsia (PE) is one of the major issues in maternal-fetal medicine. According to the American College of Obstetricians and Gynecologists (ACOG) practice bulletin, the criteria for the diagnosis of PE are new-onset hypertension (systolic/diastolic pressure ≥140/90 mmHg) and proteinuria (a 24-h urine specimen with >300 mg protein) after gestational week 20 (Author Anonymous, 2019). PE occurs in approximately 4.6% of all pregnancies and causes substantial maternal and fetal morbidity and mortality worldwide (Abalos et al., 2013; Jeyabalan, 2013), and it affects approximately 1–2% of pregnancies in Taiwan (You et al., 2018). Several pathophysiological abnormalities, such as abnormal trophoblast differentiation and invasion, placental and endothelial dysfunction, immune maladaptation, and an exaggerated systemic inflammatory response, have been proposed to explain the pathogenesis of PE (Sibai et al., 2005; Sibai, 2008). However, the underlying pathophysiology remains unclear.

PE can occur in the presence of trophoblast tissue with hydatidiform moles even in the absence of a fetus and abate with the delivery of the placenta (Lain and Roberts, 2002). This finding suggests that the placenta should be the root cause of PE, and analysis of the placenta should help to elucidate the pathophysiology of PE. Clinical and experimental findings indicate that reduced placental perfusion is a unique feature that may result in PE, and PE is commonly found with diseases involving microvascular disorders (e.g., hypertension, diabetes mellitus) or with large placentas (e.g., hydatidiform moles, multiple gestations) (Roberts and Gammill, 2005). Fibrinogen-like protein 1 (FGL1) is a protein that contains the fibrinogen-related domain in its C-terminal portion (Hara et al., 2001). Proteins structurally related to FGL1 (angiopoietins, fibrinogen, tenascins) have been implicated in multiple cellular processes, including angiogenesis, proliferation, apoptosis and extracellular matrix modulation (Procopio et al., 1999; Kim et al., 2000; Sahni and Francis, 2000; El-Karef et al., 2007), suggesting a potential role for placental FGL1 in these processes during PE progression.

FGL1, also known as HFREP1 or hepassocin, was initially identified as an enriched transcript in hepatocellular carcinoma and rodent liver regeneration (Yamamoto et al., 1993; Hara et al., 2001). The forkhead box class O (FoxO) family comprises ubiquitously expressed transcription factors, and a predicted FoxO3 binding site was found in the FGL1 gene promoter (Liu et al., 2018). Increased plasma FGL1 levels disrupt insulin signaling to induce insulin resistance and type 2 diabetes through an extracellular signal-regulated kinase 1/2-dependent pathway. Accordingly, FGL1 knockdown improved insulin resistance in both high-fat-diet-fed mouse and ob/ob mouse (Wu et al., 2016). Our previous study indicated that FGL-1 was mainly expressed by placental trophoblasts and functions to increase trophoblast proliferation (Kang et al., 2020). However, limited data are available regarding the role and regulatory mechanisms of FGL1 in PE and PE-associated disorders.

Treating pregnant rodents with NG-nitroarginine methyl ester (L-NAME), a nitric oxide synthase (NOS) inhibitor, is an accepted method of inducing PE, and the experimental outcomes are similar to those in human PE, including hypertension, proteinuria, renal damage, and intrauterine growth restriction (Yallampalli and Garfield, 1993; Molnar et al., 1994; Buhimschi et al., 1995; Tsukimori et al., 2008). In this study, trophoblast cell lines, an L-NAME-induced PE-like mouse model, and human placental samples were used to evaluate the role of FGL1 in the progression of PE and the possible related regulatory mechanisms. Because FGL1 has been implicated in liver injury as a mitogenic growth factor (Cao et al., 2011), reduces the expression of proapoptotic factors (Li et al., 2010), and facilitates damaged liver tissue regeneration (Yan et al., 2002), we hypothesized that upregulated FGL1 expression may function as a complementary modulator to reduce the progression of PE and verified this hypothesis by treating L-NAME-induced PE-like mouse with recombinant FGL1.

## Materials and Methods

### Materials

Mouse anti-human FGL1 monoclonal antibody, goat anti-mouse FGL1 polyclonal antibody, purified recombinant human FGL1 (the similarity of FGL1 between humans and mouse is greater than 80%), and an FGL1 enzyme-linked immunosorbent assay (ELISA) kit were obtained from R&D Systems (Minneapolis, MN, United States). Antibodies recognizing actin, cytokeratin 7 (CK7), IL-6, and PlGF were purchased from Santa Cruz Biotechnology (Santa Cruz, CA, United States), and 4′,6-diamidino-2-phenylindole hydrochloride (DAPI) was purchased from ATT Bioquest (CA, United States). Glyceraldehyde-3-phosphate dehydrogenase (GAPDH, AM4300) was purchased from Thermo Fisher Scientific (Waltham, MA, United States). Rabbit anti-human FoxO3a monoclonal antibody was purchased from Cell Signaling Technology (Danvers, MA, United States). The FGL1 expression plasmid (pCMV6- FoxO3a) was purchased from Origene (Rockville, MD). All remaining reagents, unless otherwise specified, were obtained from Invitrogen (Carlsbad, CA, United States).

### Human Study

From February 3, 2017, to July 31, 2020, in the Department of Obstetrics and Gynecology of NCKUH, we prospectively collected 38 cases of PE meeting the ACOG criteria (2019) and 42 healthy controls. PE was defined as the development of hypertension in a previously normotensive pregnant woman after 20 weeks of gestation, accompanied by new-onset proteinuria. Hypertension was defined as systolic blood pressure ≥140 mmHg or diastolic blood pressure ≥90 mmHg on at least two occasions and 4–6 h apart. Proteinuria was defined as ≥300 mg protein excretion in the 24-h urine collection or a protein concentration of ≥300 mg/L in urine (≥1 + on dipstick). Plasma samples of all participants were obtained at the time of admission for delivery. Human placentas used in this study were obtained at the end of gestation, immediately after vaginal delivery or during cesarean section. The tissues were harvested by penetrating the center of the placenta (umbilical cord insertion) and snap-frozen in liquid nitrogen, and others were fixed-embedded in paraffin for immunohistochemistry. The decidua regions were performed to RT-PCR and Western blotting. During blood pressure measurement, patients were resting in the supine position in a quiet environment, and measurements were obtained in a fasting state between 08:00 and 10:00 AM. None of the healthy controls had clinical signs of PE or other medical or pregnancy complications. The exclusion criteria were as follows: multiple pregnancies, premature rupture of membranes, chorioamnionitis, chronic hypertension, diabetes mellitus, autoimmune disorders, and fetal abnormalities.

### Cell Culture and Plasmid Transfection

The human trophoblast cell lines 3A-subE (ATCC CRL-1584) and HTR-8/SVneo (ATCC CRL-3271) were obtained from the American Type Culture Collection (ATCC, Manassas, VA, United States), cultured in growth media [high-glucose DMEM containing 10% heat-inactivated fetal bovine serum (FBS), 1% antibiotics (penicillin and streptomycin; Invitrogen), and L-glutamine (292 mg/L)] and maintained at 37°C with 5% CO_2_. For transient transfections, Lipofectamine 2000 reagent (Invitrogen) was used according to the manufacturer’s instructions. The cells were collected for further experiments after transfection with plasmids (2 mg) for 48 h. Cells (2  ×  10^5^ cells/well) were seeded in 6-well plates, and after incubation with the indicated dose of L-NAME (0, 100, 1,000 μM) for 24 h, the cells were further analysed.

### Experimental Animals and the Mouse Model of PE

Adult pregnant mouse (body weight: 21–23 g), the C57BL/6 inbred strains, were purchased from the BioLASCO Animal Center (Taipei, Taiwan, ROC) and received in our animal facilities on day 7 of pregnancy (day 0 = day of positive sperm smear). All animals were given free access to food and water. For the PE-like mouse model, L-NAME (75 mg/kg/day) was subcutaneously administered with or without recombinant FGL1 (1 μg/kg/day) on days 8–14 of pregnancy (FGL1 and non-FGL1 groups, respectively) based on a previous report (Motta et al., 2015). Both the control and treatment groups of mouse were age-matched, and saline was administered to the control groups. Blood pressure was noninvasively measured during pregnancy on gestational days (GDs) 8–20. The mouse were placed in metabolic cages for urine collection on GDs 12–15 and 15–18. The entire placenta without yolk sac and uterine layers were collected at GDs 19 for RNA isolation and Western blotting. The animal care and experimental procedures conformed to the ARRIVE guidelines (Kilkenny et al., 2010) and were approved by the Institutional Animal Care and User Committee (150213) at NCKU, Tainan, Taiwan.

### RNA Isolation and Quantitative Real-Time Reverse Transcription Polymerase Chain Reaction

Total RNA from mouse placentas was extracted using the RBC Total RNA Kit (Life Biomedical Limited) according to the manufacturer’s instructions. Next, 2 μg of extracted RNA was reverse-transcribed to cDNA using MuLV reverse transcriptase, 1 mM dNTPs, and 0.5 mg/ml oligo (dT12–18) as previously described (Cheng et al., 2009). cDNA was amplified through qRT-PCR using Fast Quant Green Master Mix with Low ROX (Protech Technology Enterprise, WI, United States) with 0.5 μM primers according to the manufacturer’s protocol. The sequences of the primers are as follows: FGL1 (F: 5′-AGC​TGC​CTG​TGT​TAT​TTC​CTC​TCA-3′; R: 5′-CCA​GGG​AGC​CAT​TTT​ATT​TAT​CCA​A-3′) (Demchev et al., 2013), interleukin (IL)-1b (F: 5′-CAC​CTC​TCA​AGC​AGA​GCA​CAG -3′; R: 5′-GGG​TTC​CAT​GGT​GAA​GTC​AAC-3′), IL-6 (F: 5′-TCC​TAC​CCC​AAC​TTC​CAA​TGC​TC-3′; R: 5′-TTG​GAT​GGT​CTT​GGT​CCT​TAG​CC-3′) (Iwashita et al., 2014), placenta growth factor (PlGF) (F: 5′-TCT​GCT​GGG​AAC​AAC​TCA​ACA-3′; R: 5′-GTG​AGA​CAC​CTC​ATC​AGG​GTA-3′) (Fu et al., 2020), FMS-like tyrosine kinase 1 (Flt-1) (F: 5′-AGG​AGA​TGC​TCC​TCC​CAA​A-3′; R: 5′-GTG​CAG​GGA​TCC​TCC​AAA​T-3′) (Zhou et al., 2007), and GAPDH (F: 5′- TGC​ACC​ACC​AAC​TGC​TTA​GC-3′; R: 5′- GGC​ATG​GAC​TGT​GGT​CAT​GAG-3′) (Xie et al., 2012). GAPDH gene expression was used as an internal control, and the $2^{-\text{ΔΔCt}}$ method was used to analyze the relative gene expression (Livak and Schmittgen, 2001).

### Histochemical Analysis

Histochemical analysis of 5-μm-thick sections was performed as described previously (Kang et al., 2020). Slides were counterstained using hematoxylin and eosin (Gill No. 1, Sigma-Aldrich), dehydrated in an ascending series of ethanol, cleared in xylene, and mounted with Cytoseal (Thermo Fisher Scientific). The center of the mouse placenta disc was marked with ink and sections were cut through the center. Photographs were obtained using an Olympus DP72 camera mounted on a BX61 Olympus microscope. The images were quantified using ImageJ software (National Institutes of Health) to analyze the spongiotrophoblast area.

### Immunohistochemistry

Cells were fixed in 2% (w/v) formaldehyde and blocked with 10% (v/v) goat serum. Sections were dewaxed and rehydrated, followed by antigen retrieval. Peroxidase method: Sections exposed to the primary antibody for 30 min at room temperature, washed in phosphate-buffered saline (PBS), incubated for 30 min with rabbit-anti-mouse IgG conjugated with horseradish-peroxidase, then staining was achieved by incubating with 3,3′-diaminobenzidine (DAB; Sigma). Fluorescence method: One percent (w/v) Sudan black in 70% (v/v) ethanol was used for 1 min to block autofluorescence. Goat serum (10%, v/v) was applied for 1 h at room temperature for nonimmune blockade, followed by overnight incubation with mouse FGL1 polyclonal antibody (Sigma, diluted 1:100) at 4°C. Goat anti-mouse secondary antibody conjugated with Alexa Fluor-488 (Invitrogen) was applied at a 1:200 dilution with DAPI nuclear counterstaining for 1 h at room temperature. The sections were mounted using Shandon Immu-Mount (Thermo Fisher Scientific). For the negative control, the primary antibody was omitted to control for autofluorescence. Slides were stored at 4°C in the dark until photographed. ImageJ was used to assess the fluorescent images to quantify the mean fluorescence intensity or area.

### ELISA

FGL1 (pg/ml) was measured in diluted human and mouse serum using an ELISA kit (R&D Systems) per the manufacturer’s protocol.

### Blood Pressure Measurement

Systolic and diastolic blood pressures were measured daily in conscious mouse by tail-cuff plethysmography using a BP-2000 blood pressure analysis system (Visitech Systems, Apex, NC, United States) in animals that had been prewarmed in a metal chamber maintained at 30°C. Each day, values obtained from three consecutive measurements were averaged. The mean arterial pressure [MAP; (2 × diastolic + systolic)/3] was calculated as previously described (Domanski et al., 1999).

### Total Urinary Proteins

Urinary protein concentrations were measured using Folin’s phenol reagent method (Lowry et al., 1951). Urine samples and bovine serum albumin standards were assayed in triplicate and read at 650 nm with an NIR filter in a spectrophotometer (model 6/20, Coleman). Sample urinary protein levels (mg/ml) were extrapolated from a standard curve derived from bovine serum albumin standards.

### Western Blot Analysis

Tissues or cells were homogenized and lysed with a buffer containing 0.1% SDS, 0.5% sodium deoxycholate, 1% NP-40, and a protease inhibitor mixture (PMSF, aprotinin, and sodium orthovanadate). Total protein lysate (50 μg per lane) was separated on SDS-PAGE gels (10% running and 4% stacking), transferred to PVDF membranes and analyzed using the designated primary antibodies (1:1000 dilution). After the membranes were probed with an appropriate secondary antibody (1:2000 dilution), signals were detected using an enhanced chemiluminescence reagent (Amersham Pharmacia Biotech, United Kingdom) under a LAS3000 imaging system (Fujifilm, Tokyo, Japan). The band intensity was quantified using ImageJ software.

### Cell Proliferation Assay

HTR-8/SVneo cells were seeded in 96-well plates (5 × 10^3^ cells/well) and cultured in DMEM containing 2% FBS for 12 h. Then, recombinant FGL1 (0, 50, 100, 200 ng/ml) was added to the culture medium, and the cells were cultured for another 48 h. Cell viability was subsequently analyzed by Cell Counting Kit-8 (CCK-8) colorimetric assays (Dojindo Molecular Technologies, Inc.). After treatment, 10 μl of CCK-8 was added to each well, and the incubation was continued at 37°C for 2 h in the dark. Then, the absorbance value at 450 nm was determined using a microplate reader (SpectraMAX 340; Molecular Devices, Sunnyvale, CA, United States).

### Statistical Analysis

All cellular experiments were performed using triplicate samples and repeated at least three times. Statistical analyses were conducted using Prism 7.0 (GraphPad Software). Data are expressed as the mean ± SEM. The D’Agostino-Pearson normality test was used to assess the normality of the distribution. Student’s *t* test was used for comparisons between the two groups. One-way analysis of variance (ANOVA), followed by post hoc analysis (Tukey’s test), was used for comparisons among multiple groups. For comparisons among multiple groups and different time points, two-way ANOVA was used. *p* < 0.05 was considered statistically significant.

## Results

### FGL1 Expression was Upregulated in Both Mouse With Experimental PE and Human Patients

**FIGURE 1:**
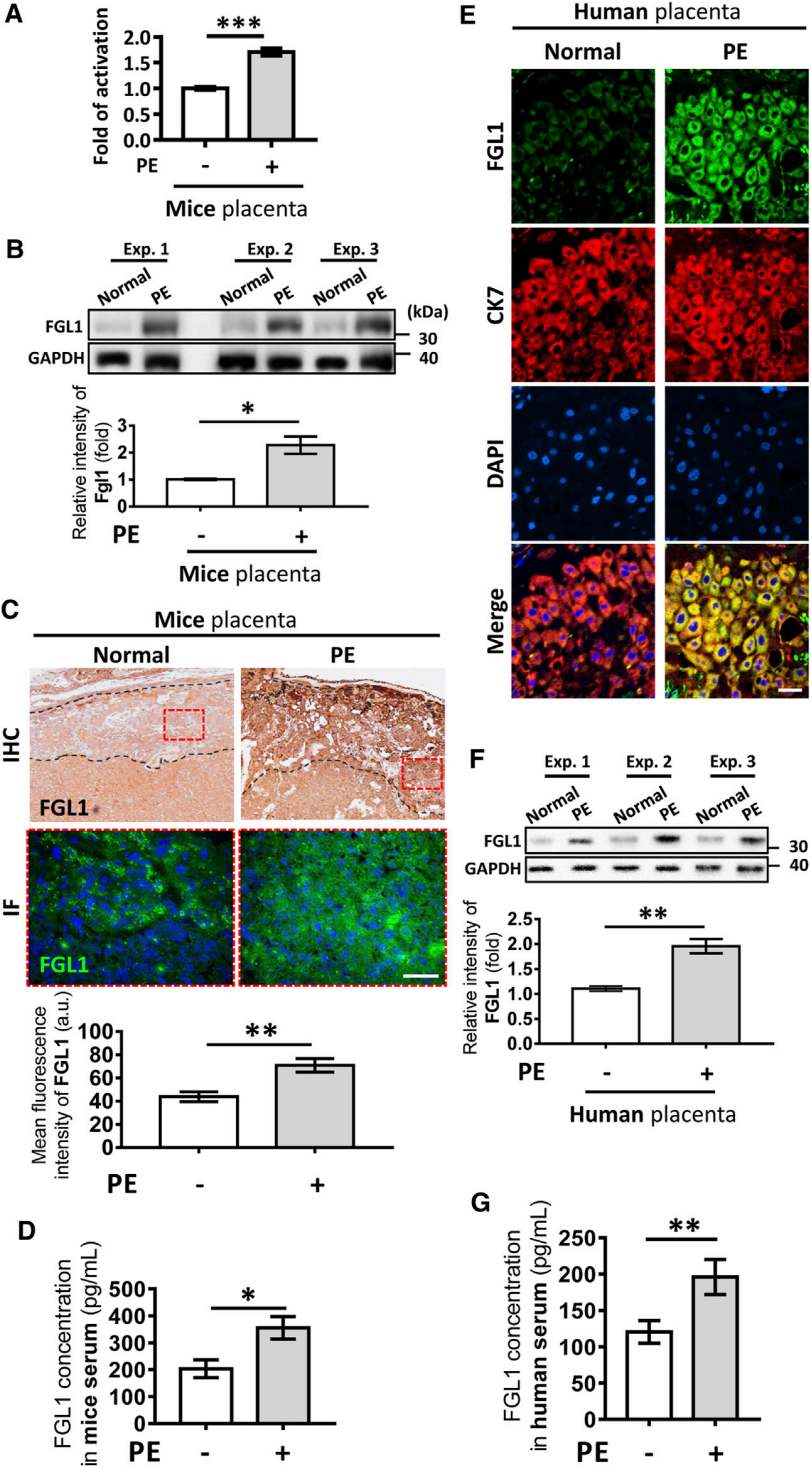
Expression of fibrinogen-like 1 (FGL1) in NG-nitroarginine methyl ester (L-NAME)-induced mouse with preeclampsia (PE) and human placenta and serum. FGL1 mRNA expression levels **(A)** and FGL1 protein expression levels **(B)** in the placenta of the mouse with PE and the controls were evaluated using RT-PCR and Western blotting, respectively. Glyceraldehyde-3-phosphate dehydrogenase (GAPDH) was used as a loading control. **(C)** FGL1 protein expression in mouse placental sections was assessed using immunohistochemistry (IHC) and immunofluorescence (IF) staining. The dotted black area indicated the decidua; the dotted red rectangle indicated the picture area of IF. Brown and green indicates FGL1; blue indicates cell nuclei. Scale bar, 200 μm. Photographs were obtained from the placenta junctional zone. **(D)** Serum FGL1 concentration was analyzed in the PE and non-PE groups using enzyme-linked immunosorbent assays (ELISAs), *n* = 6/group. **(E)** Human placental FGL1 expression in the decidua was evaluated using immunofluorescence staining. Keratin-7 (CK7) is a trophoblast cell marker, and 4′,6-diamidino-2-phenylindole (DAPI) is an ideal nuclear counterstain. Scale bar, 10 μm. **(F)** Quantified protein expression level of FGL1 in human placenta. The experiment was repeated at least three times. **(G)** Serum FGL1 concentration in pregnant women with PE (*n* = 38) and those without PE (*n* = 42). *p < 0.05; **p < 0.01; ***p < 0.001 (Student’s *t* test).

First, FGL1 gene and protein expression levels in the placentas from L-NAME-induced PE-like mouse were measured to determine the correlation between FGL1 and PE. qPCR, Western blotting, and immunofluorescence staining indicated that the FGL1 mRNA and protein expression levels in the placentas of the mouse with PE were significantly higher than those in the normal placentas (mRNA: 1.71 ± 0.07 vs. 1.0 ± 0.04-fold, *p* = 0.0001; Figure 1A; protein: 2.28 ± 0.32 vs. 1.01 ± 0.04-fold, *p* = 0.03; Figure 1B; immunofluorescence intensity: 70.94 ± 5.88 vs. 43.86 ± 4.18 a.u., *p* = 0.0038; Figure 1C). FGL1 is a secreted protein; ELISA revealed that the FGL1 concentration was higher in the serum of the mouse with PE than in that of the control mouse (355.8 ± 41.69 vs. 203.8 ± 33.25 pg/ml, *p* = 0.0172; Figure 1D). For the analysis of FGL1 levels in humans, pregnant women with PE and age-matched healthy pregnant women without PE (normotensive) were enrolled. Clinical definitions and exclusion criteria are described in the Methods section. Clinical characteristics of study subjects are shown in Table 1. As expected, patients with PE had a higher blood pressure and percent proteinuria but reduced placental weight, birth weight, and 5-min APGAR score than the healthy subjects. In our study population, the PE group had a significantly higher BMI and lower gestational age of sample collection. Trophoblast FGL1 expression in human placental sections (Figure 1E) and placental FGL1 protein levels (Figure 1F) were both significantly higher in the women with PE than in the controls. Similar results were obtained for the serum FGL1 concentration (196 ± 24.25 pg/ml, *n* = 38 vs. 120.4 ± 15.69 pg/ml, *n* = 44, *p* = 0.0088; Figure 1G). Taken together, the results suggest that the placenta trophoblasts in decidua should be one of the sources of FGL-1 production in both mice and humans and FGL1 upregulation correlates with PE.

**TABLE 1:** Clinical characteristics of study subjects with and those without Preeclampsia.

	Normotensive	Preeclampsia
Number of cases	42	38
Maternal age (y)	32.8 ± 0.6	34.2 ± 0.7
Body mass index (BMI)	25.3 ± 0.4	31.5 ± 0.9^****^
Systolic pressure (mmHg)	116.3 ± 1.2	147 ± 1.8^****^
Diastolic pressure (mmHg)	72.1 ± 1.1	91.8 ± 1.3^****^
Proteinuria (%, >30 mg/dl)	10%	49%
Placenta weight (g)	657.4 ± 25.8	538 ± 22.3^***^
Primiparous (%)	59.61%	70.21%
Multiparous (%)	40.38%	29.79%
Gestational age (w)	39.6 ± 0.2	36.4 ± 1.3^*^
Birth weight (g)	3,162 ± 63	2,626 ± 115^***^
APGAR score at 1 min	8.1 ± 0.1	8.0 ± 0.2
APGAR score at 5 min	9.6 ± 0.1	9.0 ± 0.0^*^

y, year; w, weeks; g, gram. ^*^ *p* < 0.05; ^***^ *p* < 0.001; ^****^ *p* < 0.0001, compared to the Normotensive group.

### Placental Trophoblasts are One of the Sources of FGL1 Under L-NAME Stimulation, and FGL1 Contributes to Promoting Trophoblast Proliferation

**FIGURE 2:**
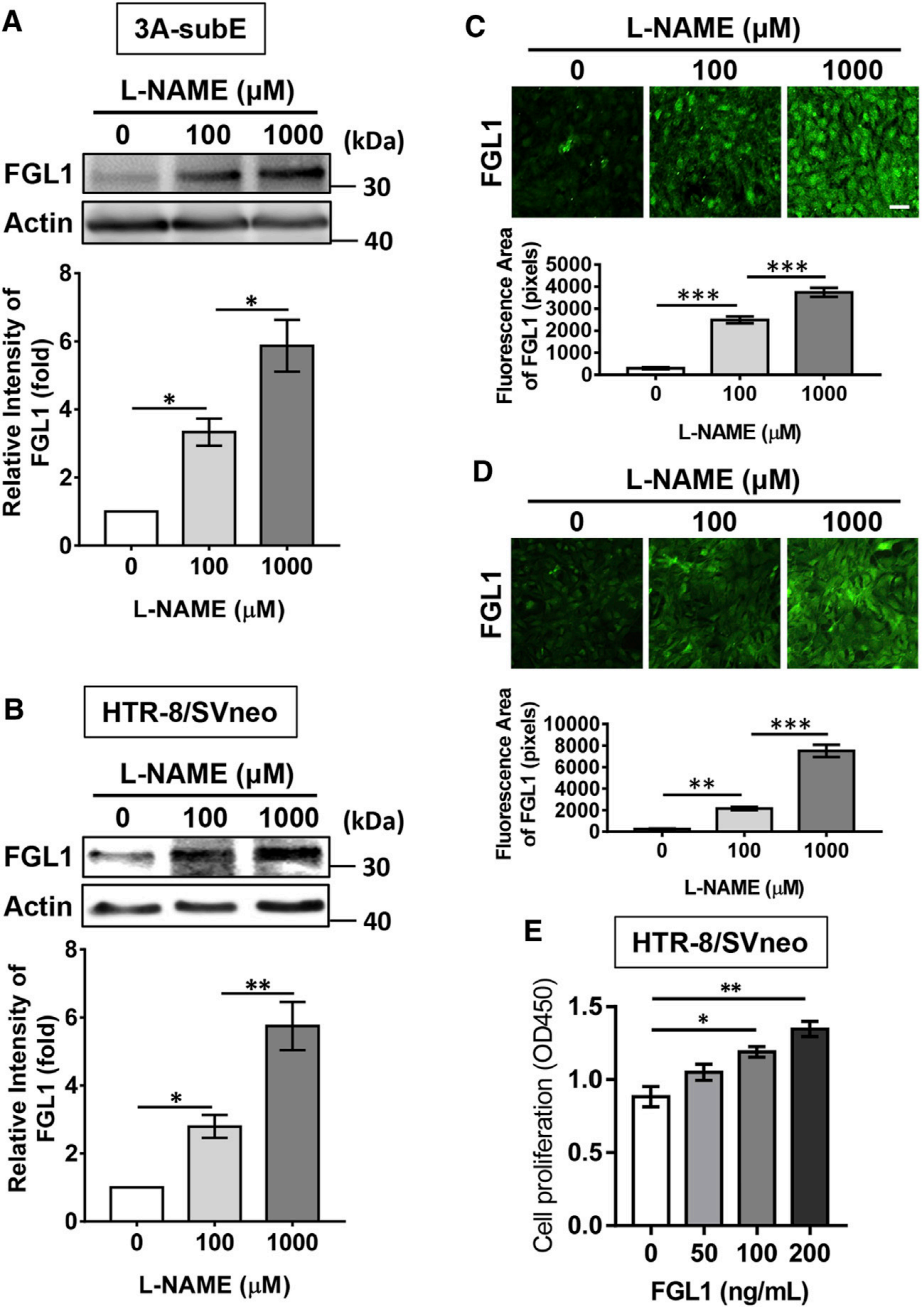
Effects of L-NAME stimulation on the expression of FGL1 in human placental trophoblast cell lines, and effects of FGL1 on the cell proliferation. Western blot analysis and immunofluorescence staining were performed to evaluate FGL1 expression in 3A-subE cells **(A,C)** and HTR-8/SVneo cells **(B,D)** incubated with the indicated dose of L-NAME for 24 h. Scale bar, 50 μm. **(E)** After FGL treatment at the indicated dose for 48 h, cell proliferation was assessed by CCK-8 assays. The experiments were repeated at least three times. *p < 0.05; **p < 0.01; ***p < 0.001 (Tukey’s test).

Our previous study indicated that FGL1 can be expressed in human trophoblasts (Kang et al., 2020). Here, we investigated whether L-NAME-enhanced placental FGL1 expression was correlated with trophoblasts. Two human trophoblast cell lines, 3A-sub E and HTR-8/SVneo, were stimulated with L-NAME at the indicated dose. As shown in Figure 2, Western blotting and immunofluorescence staining both indicated that FGL1 expression was dose-dependently upregulated under L-NAME stimulation, suggesting that L-NAME-induced progression of PE may also promote FGL1 expression in trophoblasts. We further found that exogenous FGL1 increased trophoblast proliferation (Figure 2E).

### L-NAME-Induced Progression of PE in Mouse was Reduced by FGL1 Treatment

**FIGURE 3:**
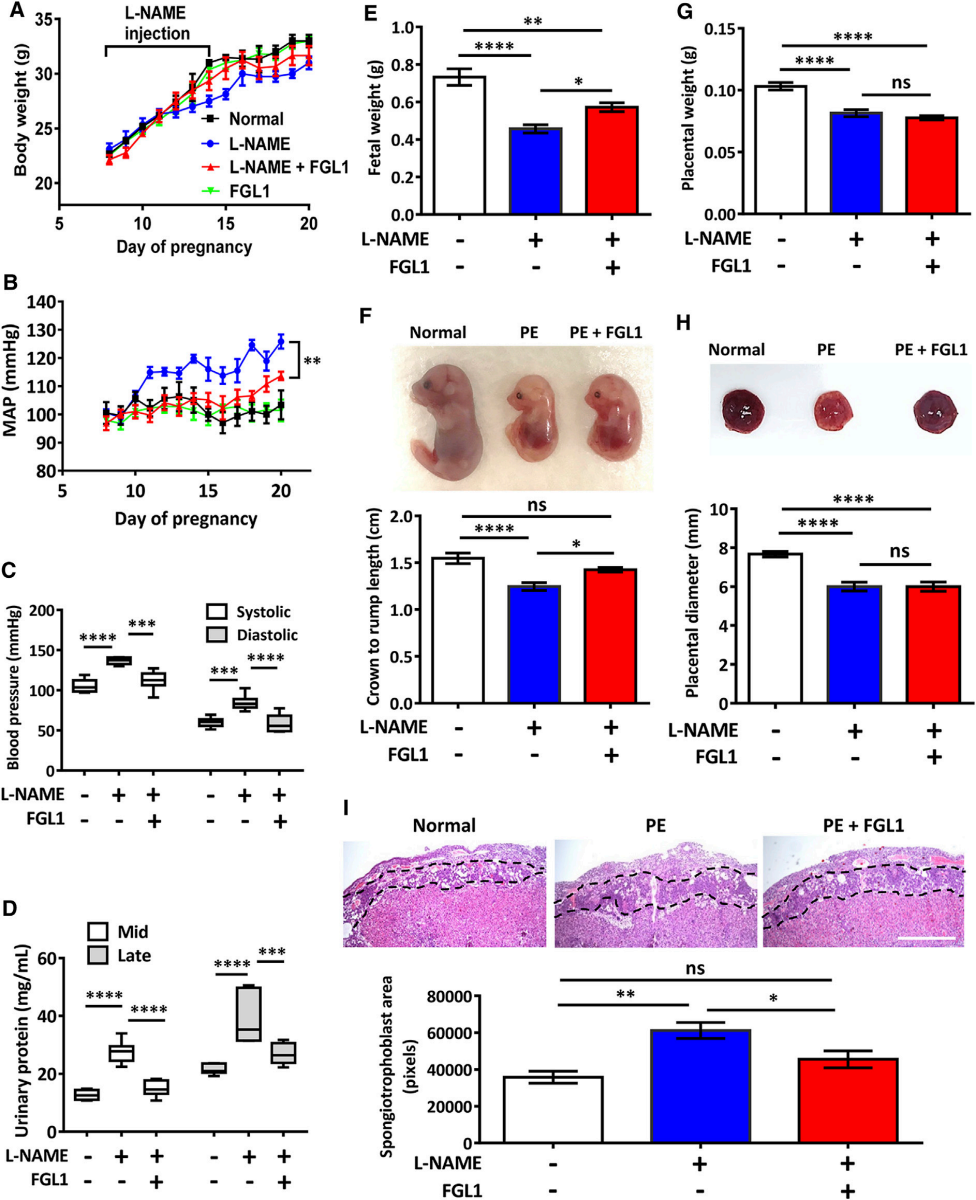
Effects of FGL1 treatment on the PE mouse. After the injection of L-NAME with or without FGL1 into pregnant mouse, representative parameters, including body weight **(A)**, mean arterial pressure (MAP) **(B)**, blood pressure **(C)**, urinary protein **(D)**, fetal weight **(E)**, crown-to-rump length of the fetus **(F)**, placenta weight **(G)**, and placenta diameter **(H)**, were analysed. Box and whisker plots for blood pressure and urinary protein. The box represents the median and interquartile range (IQR), and the whiskers were generated using Tukey’s method (values up to 75th percentile +1.5 IQR). **(I)** Hematoxylin-eosin staining of the mouse placenta. Dotted line indicates the spongiotrophoblast area. Mid, 12–15 days of pregnancy; late, 15–18 days of pregnancy. Scale bar, 10 mm. *n* = 6/group. *p < 0.05; **p < 0.01; ***p < 0.001; ****p < 0.0001; ns, no significance [**(A, B)**: two-way ANOVA test; **(C–I)**: Tukey’s test].

To determine whether the highly expressed FGL1 functioned as a protective factor against the progression of PE in mouse, we administered recombinant FGL1 to the mouse with PE. The body weight results revealed that the experimental group (PE + FGL1) had a minor improvement in body weight compared with the PE group during pregnancy (Figure 3A). Furthermore, FGL1 treatment significantly improved the MAP (Figure 3B), diastolic pressure and systolic pressure in the FGL1 group compared with the non-FGL1 group (diastolic: 58.61 ± 4.61 vs. 84.6 ± 3.95 mmHg, *p* < 0.0001; systolic: 112.28 ± 4.91 vs. 136.65 ± 1.73 mmHg, *p* = 0.0002; Figure 3C). The upregulated urinary protein expression under L-NAME stimulation at mid- and late pregnancy was significantly reduced by FGL1 treatment (mid: 14.93 ± 1.11 vs. 27.53 ± 1.57 mg/ml, *p* < 0.0001; late: 26.89 ± 1.47 vs. 38.93 ± 3.62 mg/ml, *p* = 0.0002; Figure 3D; *n* = 6/group). The results in this section suggest that upregulated FGL1 expression has critical antihypertensive and antiproteinuric effects in the mouse with PE, which may inhibit the progression of PE.

Next, the effects of FGL1 treatment on various parameters related to the fetus and placenta were investigated. Measurement of fetal weight and crown-to-rump length revealed that FGL1 treatment significantly reversed the L-NAME-reduced fetal growth (fetal weight: 0.57 ± 0.02 vs. 0.46 ± 0.02 g, *p* = 0.038; Figure 3E; crown-to-rump length: 1.43 ± 0.03 vs. 1.25 ± 0.04 cm, *p* = 0.033; Figure 3F; *n* = 12/group) but did not significantly affect placental weight or diameter (placental weight: 0.08 ± 0.002 vs. 0.08 ± 0.003 g, *p* = 0.564; Figure 3G; diameter: 6.0 ± 0.242 vs. 6.0 ± 0.229 cm, *p* > 0.99; Figure 3H; *n* = 12/group). In addition, hematoxylin-eosin staining performed to analyze the placental structure revealed that the L-NAME-increased spongiotrophoblast area was significantly reduced by FGL1 treatment (45,549 ± 4,585 vs. 61,232 ± 4,333 pixels, *p* = 0.046; Figure 3I; *n* = 6/group). These results indicate that FGL1 contributes to improving fetal growth and placental structure, thus retarding the progression of PE. Thus, upregulated FGL1 expression should function as a compensation factor to reduce the progression of PE.

### FGL1 Downregulated Proinflammatory Cytokine Expression and Ameliorated the Imbalance of Angiogenic Molecules in Mouse With PE

**FIGURE 4:**
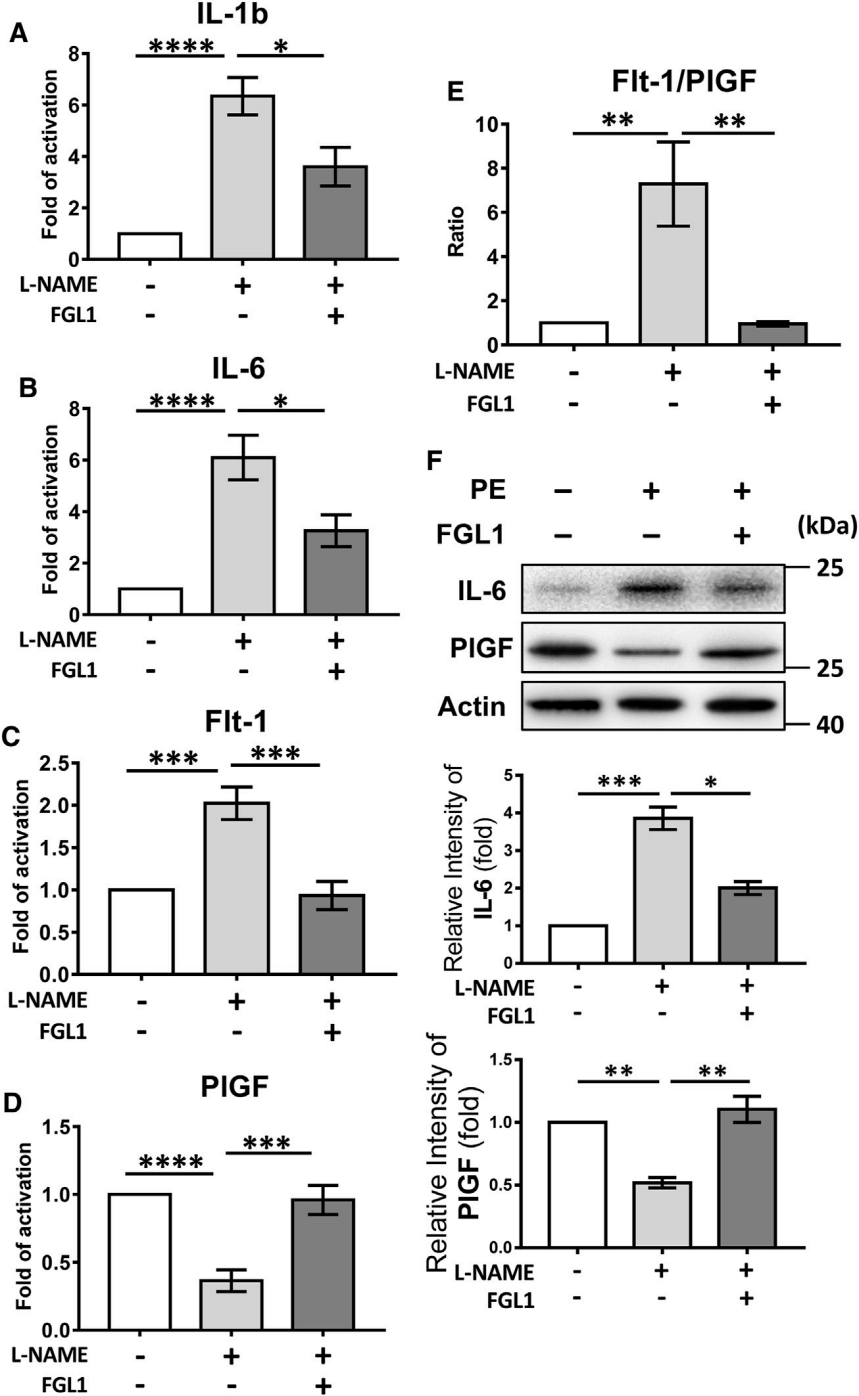
Effects of FGL1 treatment on proinflammatory cytokines and angiogenic markers in placentas from mouse with PE. After injection of L-NAME with or without FGL1, the placenta on the 18th day of pregnancy was subjected to quantitative real-time RT-PCR (qPCR) to evaluate the mRNA expression of proinflammatory cytokines and angiogenic markers. **(A)** Interleukin (IL)-1b. **(B)** IL-6. **(C)** FMS-like tyrosine kinase 1 (Flt-1). **(D)** Placental growth factor (PlGF). **(E)** Flt-1/PlGF ratio of mRNA level, *n* = 6/group. **(F)** Protein levels of IL-6 and PlGF. The experiments were repeated at least three times. *p < 0.05; **p < 0.01; ***p < 0.001; ****p < 0.0001 (Tukey’s test).

Furthermore, qRT-PCR was used to elucidate how FGL1 treatment inhibited the progression of PE in mouse. The results revealed that FGL1 treatment significantly inhibited the expression of the proinflammatory cytokines IL-1b and IL-6 under L-NAME stimulation (IL-1b: 3.60 ± 0.75- vs. 6.34 ± 0.73-fold, *p* = 0.015, Figure 4A; IL-6: 3.26 ± 0.62- vs. 6.10 ± 0.87-fold, *p* = 0.014, Figure 4B; *n* = 6/group). The imbalanced expression of the angiogenic factors Flt-1 and PlGF was also improved in the FGL1 group compared with the non-FGL1 group (Flt-1: 0.93 ± 0.17- vs. 2.02 ± 0.19-fold, *p* = 0.0003, Figure 4C; PlGF: 0.96 ± 0.11- vs. 0.37 ± 0.08-fold, *p* = 0.0002, Figure 4D; Flt-1/PlGF ratio: 0.95 ± 0.09- vs. 7.29 ± 1.91-fold, *p* = 0.003, Figure 4E; *n* = 6/group). The protein levels of IL-6 and PlGF also showed consistent results (Figure 4F). These findings indicate that FGL1 inhibits the progression of PE by reducing the expression of proinflammatory cytokines and rescuing the dysregulated levels of angiogenic molecules.

### FoxO3a Overexpression Abolishes L-NAME-Induced Upregulation of FGL1

**FIGURE 5:**
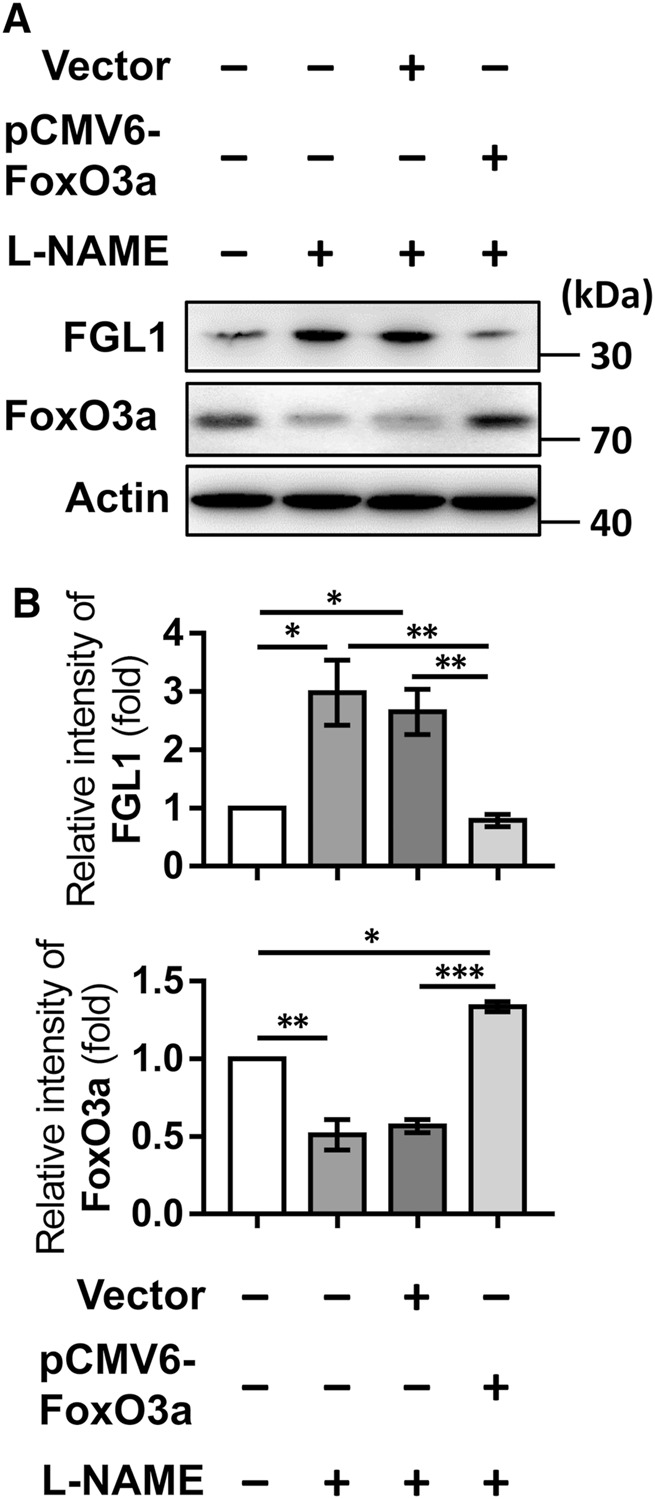
Overexpression of FoxO3a inhibits L-NAME-induced FGL1. **(A)** After transfection for 48 h and L-NAME (100 μM) treatment for 24 h, the effects of FoxO3a overexpression on L-NAME-upregulated FGL1 expression were evaluated by Western blotting. **(B)** Quantified protein expression levels of FGL1 and FoxO3a in HTR-8/SVneo cells. *p < 0.05; **p < 0.01; ***p < 0.001 (Tukey’s test).

Finally, the intracellular mechanisms by which L-NAME moderates placental FGL expression and the cell function of FGL1 were investigated. Digital prediction by Qiagen (Hilden, Germany) indicated that the transcription factor binding site of FoxO3a is contained in the FGL1 promoter. L-NAME-induced upregulation of FGL1 was accompanied by a decrease in FoxO3a, and the FGL1 increase was abolished under FoxO3a overexpression (Figures 5A,B). Here, the results suggest that the overexpression of FoxO3a inhibits FGL1 expression in L-NAME-induced trophoblast cells.

## Discussion

**FIGURE 6:**
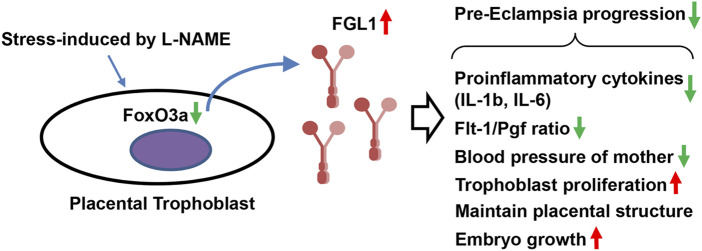
Schematic representation. Stress-upregulated FGL1 expression, through FoxO3a reduction, may function as a compensatory factor that contributes to reducing the progression of PE by mediating trophoblast proliferation, proinflammatory cytokines, and angiogenesis-related biomarkers.

In this study, we found that the gene and protein levels of FGL1 were both significantly upregulated in the placenta and serum of mouse and humans with PE, indicating a correlation between PE and FGL1. In conclusion, this study highlighted the role of placental FGL1 in PE. Stress from reduced NOS activity and impaired NO formation/bioavailability increased placental FGL1 release in trophoblasts, at least in part, through FoxO3a signaling. FGL1 may function as a complementary factor to reduce the progression of PE by promoting trophoblast proliferation, downregulating proinflammatory cytokine expression and balancing placental perfusion (Figure 6).

The L-NAME-induced PE-like mouse model has been widely used for studying disease pathogenesis, such as blood vessel injury, inflammatory factors, and molecular mechanisms (Yallampalli and Garfield, 1993; Shu et al., 2018). Because the placenta is formed at GD 10.5 and is the root cause of PE (Lain and Roberts, 2002; Sones and Davisson, 2016), based on a previous report (Motta et al., 2015), we treated mouse with L-NAME and FGL1 on GDs 7–14. The results of blood pressure and urinary protein analysis showed that FGL1 treatment significantly reduced L-NAME-induced hypertension and proteinuria, which is consistent with our hypothesis.

Studies have suggested that L-NAME-induced placental hypoperfusion and ischemia are associated with severe PE (Wang et al., 2009). Our *in vitro* study on human trophoblast cell lines demonstrated that FGL1 expression can be dose-dependently enhanced by L-NAME stimulation. L-NAME is an NOS inhibitor (Fantel et al., 1997), implying that placental FGL1 expression may be mediated through reduced NOS activity and impaired NO formation/bioavailability.

Data on the role of placental FGL1 in PE have remained limited. FGL1 is known to regulate the expression of proliferative factors, promote liver regeneration, and repair liver damage (Yan et al., 2002; Li et al., 2010; Nayeb-Hashemi et al., 2015). FGL1 was suggested to be a biomarker for epithelial-mesenchymal transition and angiogenesis in specific lung adenocarcinomas (Bie et al., 2019). These findings can be extrapolated to indicate that upregulation of placental FGL1 expression is protective against the progression of PE. Consistent with this hypothesis, our investigation revealed that recombinant FGL1 administration to the mouse with PE significantly ameliorated maternal PE parameters, inducing, for example, decreases in blood pressure and urinary protein. FGL1 is a circulating factor that could be used as a biomarker for predicting disease activity and prognosis (Liu et al., 2020). However, to date, studies on FGL1 in the vasculature are lacking, and this issue may warrant further investigation.

The placenta is the root cause of the intrauterine growth restriction observed in PE (Roberts and Escudero, 2012). Insufficient invasion of trophoblast cells into the myometrial portions of the spiral arteries is crucial for the development of PE, leading to the failure of uteroplacental vessel adaptive changes (Kaufmann et al., 2003). Consistently, our results revealed that FGL1 treatment in L-NAME-stimulated mouse improved the proportion spongiotrophoblast layer and fetal growth but did not affect the L-NAME-reduced placental diameter. These observations indicate that the mechanisms of FGL1 in the placenta may involve the regulation of blood flow and angiogenesis, thus contributing to fetal growth.

FGL1 has been suggested as a novel biomarker for angiogenesis in patients with lung adenocarcinoma (Bie et al., 2019). Additionally, angiogenic imbalance is believed to contribute to PE. An increase in the levels of some antiangiogenic factors released by the placenta into the maternal circulation, such as Flt-1, and a decrease in the levels of proangiogenic factors, such as PlGF, cause angiogenic imbalance and contribute to the pathogenesis of PE (Dymara-Konopka et al., 2018). Accordingly, our data indicated that FGL1 treatment may retard the progression of PE by reducing the expression of proinflammatory cytokines and preventing the imbalance of angiogenic factors. Our findings may contribute to further knowledge regarding the role of placental FGL1 in PE.

Our study has some limitations. First, we did not have an FGL1 gene knockout mouse strain (FGL1-KO) (Bie et al., 2019) to evaluate the effects of FGL loss on the progression of PE. Second, although the results indicated the effects of FGL1 on the proliferation of trophoblasts, performing a toxicity study of FGL1 in trophoblasts would have provided further evidence for its use as a therapeutic agent. Third, though the results of our pilot study are consistent with an increase in placental expression and serum levels of FGL2 in patients with PE, some limitations must be kept in mind. The PE group in our study population had significantly higher BMI and lower gestational age of sample collection. However, using the L-NAME-induced PE-like mouse model, we confirmed that FGL1 treatment could reduce disease progression by decreasing proinflammatory cytokines and maintaining the balance of angiogenesis, which is a strength of our study. The results in this report indicated the benefits of FGL1 treatment on mice PE progression.

Accumulating evidence has shown that FoxO3a is involved in mediating various biological processes, including proliferation, apoptosis, protection against oxidative stress, and metabolism (van der Vos and Coffer, 2011). The polymorphisms of FoxO3 on rs2232365 are associated with the risk of late-onset PE in pregnant women (Pan et al., 2020). Increased expression levels of FoxO3a were found in patients with PE, and decreasing FoxO3a expression by siRNA reduced trophoblast apoptosis (Zhang Z. et al., 2020). FoxO3a plays a role in the regulation of trophoblast development and pregnancy complications, and a decreased expression of FoxO3a was found in the placenta of PE cases and was present in trophoblasts (Chen et al., 2021). Furthermore, FoxO3a posttranslational modifications were increased by accumulated reactive oxygen species (ROS) under oxidative stress (Zhang H. et al., 2020). Therefore, these results could be an indirect result of this cellular stress induced by L-NAME and more easily controlled with anti-free radical agents such as vitamins C and D. Here, our results showed that the FoxO3a protein level was downregulated in trophoblasts under NOS inhibition. In addition, the function of FGL1 in promoting trophoblast proliferation was similar to that in our previous report (Kang et al., 2020). However, the detailed mechanisms need to be further investigated.

In conclusion, FGL1 may function as a complementary factor to reduce the progression of PE by promoting trophoblast proliferation, downregulating proinflammatory cytokine expression and balancing placental perfusion, and it might also be a prognostic marker for PE. The effects of FGL1 are at least in part mediated through FoxO3a signaling.

## Data Availability

The original contributions presented in the study are included in the article/Supplementary Material, further inquiries can be directed to the corresponding author.
